# Valorization of By-Products from White Cabbage (*Brassica oleracea* var. *capitata*) Processing

**DOI:** 10.3390/foods15061009

**Published:** 2026-03-12

**Authors:** Andra Dubrovska, Ruta Galoburda, Zanda Kruma, Liene Ozola, Evita Straumite

**Affiliations:** Food Institute, Faculty of Agriculture and Food Technology, Latvia University of Life Sciences and Technologies, Riga Street 22, LV-3004 Jelgava, Latvia; pt19012@lbtu.lv (A.D.); zanda.kruma@lbtu.lv (Z.K.); evita.straumite@lbtu.lv (E.S.)

**Keywords:** cabbage processing by-products, antioxidant activity, candied fruit syrup, jelly, texture

## Abstract

This study aimed to valorize by-products from cabbage processing to produce nutrient-rich powders that are suitable for food incorporation and, as a case study, to evaluate their application in texture-modified jelly intended for senior consumers. Freeze-dried powders from cabbage leaves and cores were analyzed for physicochemical properties, nutritional value, and antioxidant activity. Steaming significantly affected water absorption, solubility, and color: powders from fresh cabbage exhibited higher water solubility and lighter, greener hues, whereas powders from steamed cabbage showed darker, yellow–red tones due to pigment degradation. Nutritional analysis confirmed high dietary fiber contents (>30 g/100 g dry weight) in all powders. Core powders contained more potassium and phosphorus, with minimal mineral losses being observed after steaming. Sugar profiling showed greater fructose, glucose, and total sugar contents in leaf powders, whereas sucrose predominated in core powders. Steaming facilitated maltose formation. Although steaming generally reduced total phenolic content, it increased antioxidant activity in steamed leaf powders. Application trials demonstrated that cabbage powder concentrations strongly influenced jelly composition, including dietary fiber, total phenolic content and mineral levels, while pectin concentration primarily affected texture. Optimized formulations yielded nutritionally enriched jellies with acceptable sensory properties, demonstrating the feasibility of using cabbage processing by-products as a value-added food ingredient.

## 1. Introduction

During fruit and vegetable processing, not all parts of the raw materials are utilized. Portions are removed during washing and trimming, while skins, peels, and cores remain after ingredient preparation. These unused fractions are commonly referred to as fruit and vegetable by-products. In the food industry, by-products are defined as secondary materials generated during processing that are not the primary target of production but still contain valuable nutritional and functional components [1]. They are rich in dietary fiber, bioactive compounds, and minerals and therefore represent a valuable resource rather than waste. They can be recovered and reused in food systems, thereby reducing waste and supporting sustainable production [2]. Driven by various waste reduction initiatives and circular economy objectives—such as the United Nations Sustainable Development Goals, the Farm to Fork Strategy, and the European Green Deal—researchers are actively exploring the potential use of food by-products in the development of new food products [3,4,5,6].

White cabbage (*Brassica oleracea* var. *capitata*) is a traditionally cultivated plant in Europe and is widely grown and consumed in various food products, such as sauerkraut, marinated cabbage, coleslaw, and stewed cabbage. White cabbage is a nutritionally valuable cruciferous vegetable; raw cabbage typically contains about 2.5 g/100 g of dietary fiber [7], along with micronutrients such as potassium, calcium, and vitamin C, and bioactive compounds, including glucosinolates and polyphenols, that contribute to the antioxidant activity [8,9]. During cabbage processing, significant amounts of by-product are generated, mainly as outer leaves and cores; these can constitute a considerable percentage of the total raw material, depending on trimming and processing practices. Although cabbage cores are often discarded due to their dense structure, they are edible and contain nutrients similar to those in the leaf tissues.

Cabbage processing by-products are rich in dietary fiber, minerals, vitamins, prebiotic sugars, glucosinolates, isothiocyanates, and other bioactive compounds, providing strong antioxidant activity and notable antimicrobial potential [8]. Various strategies have been explored to valorize these by-products. These include the extraction of sunlight-enhanced flavonol accumulation [10] and many other bioactive compounds [5]. Another approach proposed by Zhang et al. [3] includes the production of high-value products through multi-stage processes involving low-temperature extraction, medium-temperature pyrolysis, and high-temperature activation. Cabbage waste meal has also been investigated as an alternative feed for grower-finisher pigs, showing benefits for growth performance, carcass quality, animal health, and economic returns [11]. Moreover, there is increasing interest in using cabbage waste in food formulations. For example, Bas-Bellver et al. [4] demonstrated that partially replacing rice flour with cabbage waste powder enhanced the antioxidant capacity and nutritional value of gluten-free bakery products, particularly breadsticks. Similarly, Althawab et al. [12] reported that cabbage outer leaf powder, rich in bioactive compounds and dietary fiber, improved the nutritional, antioxidant, physicochemical, and sensory properties of set-type yogurt.

One of the most practical approaches for utilizing food by-products and extending their shelf life is drying, which enables their conversion into stable powder-form ingredients. Several drying techniques have been applied to cabbage by-products, including hot air-drying at 60 °C for 8 h [8], air-drying at 30 °C for 96 h [11], and freeze-drying (lyophilisation) [12]. Freeze-drying has been shown to better preserve the nutritional and bioactive quality of products, because it removes water under low-temperature and vacuum conditions, minimizing thermal degradation [13]. This method also helps maintain natural color, flavor, texture, and structural integrity, producing a powder with higher retention of bioactive compounds, improved solubility and rehydration properties [14]. However, freeze-drying involves longer processing times and higher energy consumption and requires specialized equipment, which can increase cost and limit scalability [15]. Despite these limitations, freeze-drying is particularly suitable when the aim is to produce a high-quality functional ingredient.

Prior to drying, thermal pretreatments such as blanching, steaming, or cooking are commonly applied to vegetables to improve palatability, soften plant tissues, reduce enzymatic activity, enhance drying efficiency, and lower microbial load. However, these treatments may also affect the material’s nutritional composition. Quantitative studies show that thermal processing can cause significant changes in both bioactive and nutrient components: for example, steaming and other cooking methods can reduce water-soluble nutrients such as vitamin C and total phenolic content in *Brassica* vegetables, where losses of ascorbic acid and phenolics have been widely observed during steaming treatments compared to raw samples [16]. Several studies have reported that steaming can significantly increase the extractable phenolic compounds, likely due to the disruption of cell wall structures and improved release of bound phenolics [17].

Alongside advances in sustainable food systems, the world is also experiencing a rapid increase in the elderly population. The United Nations (UN) estimates that by 2050, the number of people aged over 65 will nearly double, while the population aged over 80 will triple, meaning that one in six people will be over 65 years old [18,19]. This demographic shift underscores the importance of improving the quality of life for older adults and reducing the risk of chronic diseases. Aging is associated with physiological changes, weakened immunity, and an increased risk of malnutrition. For some older adults, insufficient nutrition may result from dysphagia or oral health conditions that make chewing or swallowing food difficult. Reduced appetite, illnesses, medications, and impaired nutrient absorption further exacerbate nutritional inadequacy. While dietary supplements are often prescribed to correct deficiencies such as inadequate protein intake, their digestibility and nutrient bioavailability indicate that supplementation alone may not always be sufficient to ensure optimal nutritional status [20]. Therefore, there is a growing need to develop food products that are not only nutritionally balanced and rich in essential nutrients but also sensorially appealing to older adults. In this context, the utilization of fruit and vegetable by-products offers a promising approach for creating functional, sustainable, and nutrient-dense foods tailored to the specific nutritional needs of the aging population.

To reduce the generation of underutilized by-products and enhance resource efficiency, it is essential to investigate their nutritional composition and potential technological applications. However, research focusing specifically on by-products from cabbage processing and their valorization in food systems remains limited. A comprehensive understanding of their functional properties could support their use in food products intended for older adults, particularly those requiring texture modification. Therefore, this study aimed to valorize by-products from cabbage processing to produce nutrient-rich powders that are suitable for food applications. The powders were characterized in terms of nutritional composition, antioxidant activity, water absorption capacity, and color. Furthermore, their applicability was evaluated as a case study in a texture-modified jelly intended for senior consumers.

## 2. Materials and Methods

### 2.1. Cabbage Powder Production

**Figure 1:**
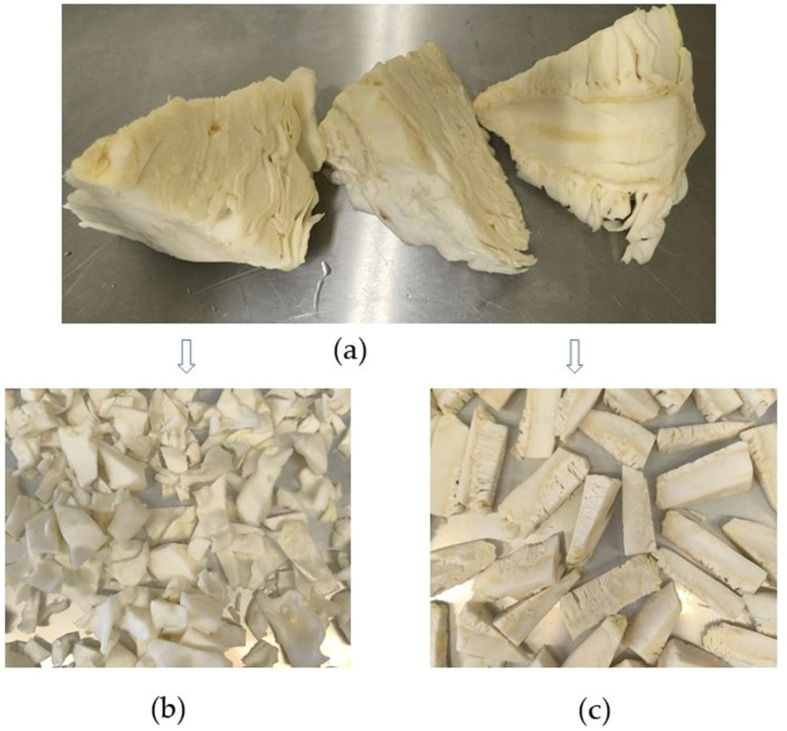
The central part of the cabbage (**a**) and its parts after cutting: leaf parts surrounding the cabbage core (**b**) and cabbage cores (**c**).

The central part of the cabbage, known as the core, along with the surrounding leaves (Figure 1a), was washed with water and briefly left to air-dry. The leaves were mechanically separated from the core. The leaves were then cut into smaller strips, while the core was diced into cubes (approximately 2 × 2 × 3 cm). Cabbage (*Brassica oleracea* var. *capitata*) processing by-products were obtained from the farms “Undzeni” (56°52′ 12″ N, 23°76′20″ E; Lielvircava, Latvia) and LLC “Dimdini” (57°19′42″ N, 26°37′76″ E; Lizums, Latvia).

The prepared samples were divided into two groups. One group was frozen fresh using a blast freezer, Sagi DF51-0P14 (Angelo Po Grandi Cucine S.p.A., Ascoli Piceno, Italy), while the second group was steamed prior to freezing. Steaming was performed using a Philips HD9126/90 steam cooker (Philips N.V., Jiaxing, Zhejiang Province, China), with the cabbage cores and leaves being processed separately. Samples were placed in three vertically stacked sections (3–4 cm layer). The cabbage leaves were steamed for 60 min, and the cores for 90 min, until softened.

Following steaming, the samples were cooled on trays and subsequently placed in a blast freezer, Sagi DF51-0P14 (Angelo Po Grandi Cucine S.p.A., Ascoli Piceno, Italy), for 1 h until the temperature reached −18 °C. The core samples were frozen for 60−90 min. Fresh samples were frozen for the same duration as the steamed ones. After freezing, all samples were placed in low-density polyethylene (LDPE) freezable zip-lock bags and stored in a freezer at −18 °C until further processing by freeze-drying.

Freeze-drying was performed using the FrostX 10 freeze-drier (FrostX Sp. z o.o., Gliwice, Poland). The cabbage leaves and cores were evenly spread on drying trays. The freeze-drying process consisted of three stages: (1) freezing at −40 °C, which lasted 3 h; (2) lyophilization, for 18−24 h depending on the sample weight, at a pressure of 120 Pa; and (3) post-drying, for 9 h at a pressure of 70 Pa.

Following freeze-drying, the samples were ground into powder using a Knifetec KN295 mill (FOSS, Hilleroed, Denmark).

### 2.2. Experimental Design for Application of Cabbage Powder in Jelly

Table 1 presents a two-factor central composite design with five replications at the center point, including standard and run orders. The design uses the alpha value of 1.4142. Nine formulations of different concentrations of cabbage powder (Factor A: 1−6%, with central point at 3.5%) and pectin (Factor B: 1.5−2.5% with central point at 2%) were made for response evaluation. The selection of gelling agent and concentration ranges was based on a previous study, which demonstrated that pectin was more suitable than agar or gelatin, providing a texture that is easy to chew and swallow [21]. In this study, a low-methoxyl amidated pectin (SOSA, Barcelona, Spain) was used as a gelling agent. The specific pectin, “Pectin Fruit NH”, is a commercially available confectionery additive intended for food thickening and gel formation. According to the manufacturer, its ingredient composition includes the thickener amidated pectin (E440ii), stabilizer diphosphate (E450ii), dextrose and acidulant tricalcium phosphate (E341ii). For the application study, a mixture of steamed cabbage leaves (75%) and cores (25%) was used.

For the preparation of jelly, cabbage powder and pectin were incorporated at the concentrations specified in Table 1. These ingredients were mixed with Japanese quince (*Chaenomeles japonica*) syrup, obtained after candied fruit production, and water, after which the mixture was heated and subsequently boiled for 6 min. The hot solution was then poured into plastic cups with a diameter of 40 mm, filling each to a height of 20 mm, cooled, and stored in a refrigerator overnight until analysis. Each sample was prepared in duplicate (two separate batches). All samples were analyzed for moisture content, total soluble solids, pH, color, texture, total phenol content (TPC), DPPH and ABTS antiradical activity, and sensory attributes (aroma, taste, and texture).

The Japanese quince syrup (Table 2) was a commercial by-product generated during fruit maceration with sugar in candied fruit production.

### 2.3. Analytical Methods

#### 2.3.1. Chemicals and Reagents

Sodium hydroxide (NaOH), hydrogen peroxide (H_2_O_2_), hydrochloric acid (HCl), sodium carbonate (Na_2_CO_3_), sodium chloride (NaCl), disodium hydrogen phosphate (Na_2_HPO_4_), potassium chloride (KCl), potassium persulfate (K_2_S_2_O_8_), L(+) ascorbic acid (C_6_H_8_O_6_), oxalic acid (H_2_C_2_O_4_), and potassium iodide(KI) were purchased from Chempur (Piekary Śląskie, Poland). Gallic acid and Folin–Ciocalteu reagent were acquired from Scharlau (Barcelona, Spain). 2,2′-Azino-bis(3-ethylbenz-thiazoline-6-sulfonic) acid (ABTS) was purchased from Thermo Fisher Scientific (St. Louis, MO, USA). Potassium dihydrogen phosphate (KH_2_PO_4_), Trolox, and 2,2-diphenyl-1-picrylhydrazyl (DPPH) reagents were obtained from Sigma-Aldrich (Darmstadt, Germany). Starch was purchased from Carl Roth GmbH + Co. KG (Karlsruhe, Germany). Ethanol (96% vol.) was obtained from Kalsnavas elevators (Kalsnava, Latvia). Kjeltec Kjeltabs Cu/3.5 were purchased from Foss Analytical (Hilleroed, Denmark), and a total dietary fiber assay kit was obtained from Megazyme (Wicklow, Ireland). All other chemicals used were analytical grade and were purchased from Sigma-Aldrich Chemie Ltd. (St. Louis, MO, USA).

#### 2.3.2. Physicochemical Analysis

The physicochemical characteristics were measured in triplicate for each of the batches. Moisture content (%) of the cabbage powder and jellies was measured using the thermogravimetric method [22]. Briefly, approximately 2 g of the sample was dried to a constant weight at 105 ± 2 °C using a Memmert Universal Oven UF55 (Memmert GmbH + Co. KG, Schwabach, Germany). Water activity of the cabbage powder was assessed with a LabMaster-aw neo hygrometer (Novasina AG, Lachen, Switzerland). Total soluble solids (TSSs) of jellies were determined using a DR301-95 refractometer (A. Krus Optometric GmbH, Hamburg, Germany), and pH was measured using a Seven Compact pH-meter (Metler Toledo, Greifensee, Switzerland).

The water absorption index (WAI) and water solubility index (WSI) of the cabbage powders were determined following a gravimetric centrifugation method described by Puttongsiri et al. [23] and Waseem et al. [24]. Briefly, 2.5 g of cabbage powder was mixed with 35 mL of distilled water in a centrifuge tube and thoroughly shaken. Samples were centrifuged in a CM-6MT ELMI Sky Line centrifuge (ELMI, Riga, Latvia) at 2300× *g* for 15 min at room temperature 20 ± 2 °C. The wet sediment was weighed to determine WAI (g sediment/g sample). The supernatant was freeze-dried to determine the mass of soluble solids; the WSI was expressed as the percentage of the original sample mass. Measurements were performed in triplicate per batch (*n* = 6), and results are reported as mean values.

Particle size of the powders was determined using a PSA 1190 laser diffraction analyzer (Anton Paar, Graz, Austria). The instrument provided particle size distribution parameters (D10, D50, and D90) and mean particle size, calculated from at least five replicate measurements and expressed in micrometers. Data were processed using Kalliope version v.3.2.5 software (Anton Paar, Graz, Austria).

The color of both cabbage powder and jellies was determined using a ColorFlex^®^ EZ spectrophotometer (HunterLab, Reston, VA, USA). Data were acquired using EasyMatch QC version 4.98 software (HunterLab, Reston, VA, USA). Color parameters were expressed in the CIE Lab color space under the D65 standard illuminant and 10° observer angle. Powder samples were evenly distributed on the surface of a glass lens, while jellies were transferred to a specially designed glass cup with a diameter of 64 mm to ensure complete surface coverage. Color analysis was performed in five replicates for powder samples and six replicates for each jelly batch.

The firmness of jelly was evaluated using a TX.HDplus texture analyzer (Stable Microsystems Ltd., Godalming, Surrey, UK) equipped with a 30 kg load cell. A delrin radiused cylindrical probe (P/0.5R) was used to penetrate the jelly to a depth of 4 mm, and the maximum force required was recorded. The results are represented as the mean of ten repetitions.

#### 2.3.3. Determination of Proximate Composition

Protein content, sugar profile and mineral content (potassium, magnesium, and phosphorus) were determined in collaboration with the Ltd. Hamilton Baltic laboratory group (Riga, Latvia), which is accredited and certified according to international standards [25]. Protein content was measured using the Kjeldahl method with a 6.25 conversion factor according to the intra-laboratory procedures. Sugar profiles were analyzed by high-performance anion-exchange chromatography with pulsed amperometric detection (HPAE-PAD). Mineral content was determined using inductively coupled plasma optical emission spectrometry (ICP-OES).

Total dietary fiber was determined using the enzymatic–gravimetric procedure described in AOAC Official Method 985.29 [26]. Dry samples were weighed in duplicate and subjected to sequential enzymatic digestion with heat-stable α-amylase, protease, and amyloglucosidase. Total dietary fiber content was then calculated based on the mass of residue and corrected for protein and ash [27].

#### 2.3.4. Determination of Vitamin C in Cabbage By-Products

Vitamin C content was measured using the iodometric titration method. For the determination of ascorbic acid, 5 g of powder was weighed and mixed with reagents according to the procedure described by Feszterová et al. [28]. During titration, an iodine solution was added until a pale blue color appeared and remained stable for 30 s. The volume of iodine used was recorded and expressed as mg per 100 g. Each sample was analyzed in triplicate.

### 2.4. Determination of Total Phenol Content and Antioxidant Activity

#### 2.4.1. Extraction Procedure for Spectrophotometric Analysis

For extraction, depending on the material, 0.250 ± 0.001 g of cabbage powder and 2.000 ± 0.001 g of jelly sample were weighed into a glass beaker. Each sample was mixed with 80% (*v*/*v*) aqueous ethanol as the extraction solvent. The mixtures were subjected to ultrasonic-assisted extraction in an ultrasonic bath YJ5120-1 (Oubo Dental, St. Louis, MO, USA). Extraction conditions varied depending on the specific matrix—30 min at 20 °C, 40 kHz for cabbage powder [29], or 10 min at 40 °C, 35 Hz for jelly [30]. After sonication, the extracts were separated by centrifugation using a CM-6MT centrifuge (Elmi Ltd., Riga, Latvia) at 2260× *g* for 10 min and followed the procedure described by Tomsone et al. [29]. All extractions were performed in triplicate.

#### 2.4.2. Spectrophotometric Analysis

Quantification of total phenolic content (TPC) and free radical scavenging activities was performed using a Jenway 6300 spectrophotometer (Barloworld Scientific Ltd., Staffordshire, England, UK).

TPC was determined using the Folin–Ciocalteu reagent [31]. Absorbance was measured at 765 nm. A calibration curve with gallic acid (R^2^ = 0.997) was used to express the phenolic content as mg of gallic acid equivalents per 100 g of dry sample (mg GAE/100 g dw).

The antioxidant activity of cabbage powder and jelly was assessed using the 2,2-diphenyl-1-picrylhydrazyl (DPPH) radical scavenging assay according to the procedure described by Yu et al. [32]. A volume of 0.5 mL of the extract was mixed with 3.5 mL of DPPH reagent. After mixing, the mixture was incubated for 30 min, and absorbance was measured at 517 nm. DPPH radical scavenging activity was expressed as mg Trolox equivalents (TE) per 100 g of sample dry weight (mg TE/100 g dw).

The 2,2′-azino-bis(3-ethylbenzthiazoline-6-sulfonic) acid (ABTS) was used to evaluate ABTS radical cation scavenging activity using the procedure described by Re et al. [33]. Sample extract solution was prepared as described by Universa et al. [27]. Absorbance was measured at 734 nm. The ABTS radical cation scavenging activity was expressed as mg Trolox equivalents (TE) per 100 g of sample dry weight (mg TE/100 g dw).

#### 2.4.3. Determination of Volatile Compounds in Cabbage Powders

The volatile profile was determined using solid-phase microextraction coupled with gas chromatography–mass spectrometry (SPME–GC/MS). The extraction procedure followed Wu et al. [34]. Briefly, 0.3 g of the powdered sample was placed into a 20 mL headspace vial and sealed. A 50/30 μm DVB/CAR/PDMS fiber (Supelco, Bellefonte, PA, USA) was used for extraction. Samples were incubated at 60 °C for 10 min without the fiber, followed by 30 min at the same temperature with fiber exposure.

After extraction, the fiber was thermally desorbed in the GC injector. Separation was performed on a polyethylene glycol (PEG) capillary column (60 m × 0.25 mm × 0.25 μm) using a Gas Chromatograph (PerkinElmer, Inc., Shelton, CT, USA). The oven temperature was initially set at 40 °C and held for 7 min, then increased to 160 °C at 6 °C/min, followed by a rise to 210 °C at 10 °C/min, with a final holding time of 15 min. Chromatograms were processed using TurboMass version 5.3.0 software. Volatile compounds were identified by comparison with the NIST98 mass spectral library and calculated retention indexes.

### 2.5. Sensory Analysis of Jelly

To determine the overall liking of the developed jelly sensory properties (aroma, taste, and texture), evaluation was conducted with 37 potential consumers (age: 65 or older, 11 males and 26 females). Each sample, weighing approximately 30 g, was served at room temperature, in a transparent plastic cup labeled with three-digit random numbers. For each panelist, the samples were presented in a randomized order. The panelists were asked to evaluate nine formulations of jelly, providing one sample per formulation (Table 1). Since each panelist needed to evaluate nine jelly samples, the evaluation was organized in two parts—5 and 4 samples in each session, with a 30 min break between evaluations. Overall liking for a jelly’s aroma, taste, and texture was determined using a 5-point Hedonic scale (1 = very much dislike to 5 = very much like). Panelists received water for cleansing the palate between samples.

All panelists signed the consent form prior to participating in the study. The sensory analysis was approved by the Ethics Committee at the Food Institute of Latvia University of Life Sciences and Technologies.

### 2.6. Statistical Analysis

Statistical analyses of the cabbage powder samples were performed using analysis of variance (ANOVA). Differences among mean values were evaluated using Tukey’s post hoc test at a significance level of *p* < 0.05; *p* < 0.01. Different letters indicate statistically significant differences between samples.

The experimental design for the application of cabbage powder in jelly is presented in Section 2.2. The center points of the experimental design were tested in five replicates to reduce experimental errors. Based on the responses obtained from the central composite design experiments, only parameters exhibiting variations greater than 10% were included in the model calculation. The coefficients of the second-order polynomial model were estimated to describe and predict system responses. Model adequacy and statistical significance were assessed using ANOVA, including evaluation of lack of fit and pure error, while adequate precision was used to determine the reliability of the model. Optimization was performed using both numerical optimization and graphical analysis through desirability functions and response surface contour plots. The desirability function was defined to maximize fiber content, potassium content, total phenolic content (TPC), ABTS scavenging activity, and taste, while minimizing color component L* and defining color component a* in range. The firmness of the jelly was targeted to be in the range of 1.5–3.0 N [35]. Data analyses were performed using the Design-Expert 23.1. (Stat-Ease Inc., Minneapolis, MN, USA).

## 3. Results and Discussion

### 3.1. Characteristics of Freeze-Dried Cabbage Leaf and Core Powders

#### 3.1.1. Physical Characteristics of Cabbage Powders

The moisture content of the freeze-dried cabbage powders ranged from 9.47 to 13.79% (Table 3).

All freeze-dried samples are expected to exhibit very good microbiological stability, as their water activity was <0.2, well below the threshold required to support microbial growth [36]. The water activity of the steamed leaf sample (LT) did not differ from those of the fresh leaf (LS) and core (KS) powders, but the other samples were different from each other (*p* < 0.01).

The water absorption index (WAI) shows the amount of water that a dry sample can absorb. A significant difference in WAI was observed among the freeze-dried fresh cabbage powders. The WAI of sample LT was 1.2 times higher than that of sample LS, suggesting that steaming prior to freeze-drying could increase the water absorption capacity of cabbage powders. In contrast, Waseem et al. [24] reported an opposite trend. In their study, the water absorption of fresh, dried cabbage samples was 6.81%, whereas blanched samples exhibited a lower value of 6.34%. It should be emphasized, however, that the study used a different drying method, which may explain discrepancies. Nevertheless, the WAI values obtained in the current study were comparable to those presented by Waseem et al. [24].

The water solubility index (WSI) of the tested cabbage powders was high, as freeze-drying promoted the retention of water-soluble compounds and induced structural changes that enhanced powder hydration. For freeze-dried fresh cabbage powders (LS and KS), WSI was higher than for steamed samples (LT and KT). There was a significant difference between the sample groups (*p* < 0.01). For steamed samples, the WSI was lower, possibly because the amount of water-soluble compounds decreased during steaming.

The largest average particle size was for sample KS, with an average of 176 μm, D_90_ of 331 μm, and D_50_ of 141 μm. For the steamed core sample (KT), the particle sizes were smaller, with an average of 98 μm, D_90_ 217 μm, and D_50_ 61 μm. Leaf samples, LS and LT, exhibited similar particle size distributions: LS had an average of 48 μm (D_90_ 129 μm, D_50_ 24 μm), and LT had an average of 48 μm (D_90_ 118 μm, D_50_ 28 μm). Particle size distribution varied between leaf and core samples (Figure S1). This can be attributed to the softer structure and more uniform shape of the cabbage leaves, which facilitated more even grinding.

For cabbage core samples, steaming softened tissue made it easier to grind, resulting in a reduction in particle size. Bas-Bellver et al. [37] reported particle size distributions for cruciferous vegetable powders obtained by different drying methods: for cabbage samples, D_90_ was 323 μm and D_50_ 101 μm, while hot-air-dried powders had D_90_ values of 498–792 μm and D_50_ of 197–283 μm [37]. Compared to these results, the freeze-dried cabbage powders in the present study had considerably smaller particle sizes, indicating that freeze-drying produces fragile and porous structures that are easier to grind into a homogeneous powder. These observations indicate that drying method and pre-treatment strongly influence powder properties. In the current study, the comparison between KS and KT samples showed that steaming of dense core tissues prior to freeze-drying increased brittleness and porosity, facilitating the production of finer and more uniform powders.

Freeze-dried fresh cabbage leaf and core powders (LS and KS) had a lighter color than steamed freeze-dried cabbage powders (LT and KT) (Table 4). Both steamed samples were similar, but the remaining samples showed significant differences (*p* < 0.01). Regarding the green–red color spectrum (a*), fresh cabbage samples had a tendency towards a light green hue, whereas the steamed samples were close to a neutral tone, with a tendency towards a red hue. All samples were significantly different (*p* < 0.01). The blue–yellow color spectrum (b*) for all samples showed a yellow hue. Of the steamed samples, LT 15.27 ± 0.37 and KT 14.71 ± 0.65 had the most pronounced yellow color, and the data did not exhibit a significant difference, but when fresh samples were compared, LS 13.47 ± 0.18 and KS 11.93 ± 0.24, the data showed a significant difference (*p* < 0.01). When fresh (LS and KS) and steamed (LT and KT) samples were compared regarding their processing method, the fresh samples were lighter and had a light green hue, whereas the steamed samples were darker and had more yellow and red hues. Steamed samples were 1.2 times more yellow than fresh samples. Steaming degrades biologically active compounds, such as chlorophyll, which may be present in cabbage leaves and gives them their green color [38]. The yellow and red colors indicate cell degradation due to the Maillard reaction, in which carbohydrates are broken down by high temperatures and the product caramelizes or browns.

#### 3.1.2. Nutritional Value of Cabbage Powders

The total dietary fiber content did not differ significantly in fresh (KS) and steamed cabbage cores (KT), nor between steamed leaf sample (LT), fresh leaf sample (LS), and the fresh cabbage core sample (KS) (Table 5). Although there was no significant difference between fresh and steamed samples of leaves and cores, it was observed that the fiber content of steamed samples increased by approximately 1 g per 100 g dw. According to the data, the fiber content did not change significantly, and therefore it could be assumed that steaming did not significantly affect the fiber content in cabbage products. The fiber content in samples was above 30 g/100 g dw, indicating a high fiber content.

Adequate protein intake is essential for older adults to prevent the loss of muscle mass and maintain mobility [39]. Older individuals who are affected by malnutrition or sarcopenia are particularly advised to monitor their dietary protein intake, as sufficient protein consumption may help better preserve physical strength, support cardiovascular system function, and assist in body weight control [40]. In the present study, the highest protein content was determined in sample KS (16.08 ± 1.25 g/100 g dw), which did not differ significantly from sample KT (15.91 ± 1.33 g/100 g dw), indicating that steaming had no measurable effect on protein content in the cabbage core. Both leaf samples exhibited lower protein contents than the core samples. Comparable results have been reported by Waseem et al. [24], who found that fresh cabbage powder contained 12.18 g/100 g, while blanched samples contained 12.05 g/100 g, indicating minimal changes due to thermal processing. In another study, Shinali et al. [41] reported that fresh, unprocessed cabbage contained 1.28 g of protein per 100 g of raw product. These findings highlight the substantial variability in cabbage protein content, which may be attributed to differences in cultivar, processing methods, and harvest conditions. Overall, the protein contents observed in the present study fall within the range reported in previous research.

Freeze-dried fresh cabbage cores and leaves exhibited the same vitamin C contents (0.317 g/100 g dw). In contrast, the vitamin C content of freeze-dried steamed cabbage leaves and cores was approximately 1.7 times lower than that of freeze-dried fresh samples (LT and KT). This reduction may be attributed to the thermal sensitivity and water solubility of vitamin C, as exposure to steam promotes its degradation and leaching. No significant differences (*p* > 0.05) were observed between leaves and cores within the same processing group, nor between the two steamed samples. These results indicate that the vitamin C contents in cabbage processing by-products are primarily influenced by the processing method rather than by the plant fraction (leaf versus core). However, significant differences (*p* < 0.05) were found between freeze-dried fresh cabbage samples (LS and KS) and freeze-dried steamed samples (LT and KT). Previous studies report that fresh, unprocessed cabbage contains approximately 28 mg/100 g vitamin C (fresh weight basis) [42], while other data indicate a broader range of 18.8–47.0 mg/100 g fresh product in white cabbage [43]. Podsedek [43] found that ascorbic acid content may vary up to 2.5-fold among cabbage varieties, depending on genetic factors and growing conditions. Similarly, Tanongkankit et al. [44] reported vitamin C contents of 619.85 mg/100 g dw in fresh cabbage and 626.60 mg/100 g dw in blanched cabbage, indicating that the effects of processing may differ depending on treatment conditions. Overall, the literature suggests considerable variability in cabbage’s vitamin C content due to cultivar, season, environmental conditions, and processing methods. According to the European Parliament and Council Regulation (EU) No. 1169/2011 (25 October 2011) on the provision of food information to consumers [45], a product or ingredient may be considered a significant source of vitamin C if it provides 15% of the nutrient reference value, which is 80 mg per 100 g of product. Based on this criterion, the analyzed cabbage powders can be regarded as a substantial source of vitamin C. Since the human body cannot synthesize vitamin C (ascorbic acid), it must be obtained through the diet. Vitamin C acts as a potent antioxidant, neutralizing free radicals and thereby protecting cells from oxidative damage [28]. It is essential for collagen synthesis, contributing to the formation of bones, skin, blood vessels, and cartilage. Additionally, it improves iron absorption and supports immune function [46]. Although vitamin C deficiency is uncommon in individuals consuming a balanced diet, evidence suggests that adequate vitamin C intake may help reduce the risk of Alzheimer’s disease and potentially mitigate age-related muscle degeneration [47].

Minerals play an essential role in the human body, as they are involved in numerous vital physiological functions. Potassium contributes to blood pressure regulation, supports proper nervous system function, and contributes to muscle function. Magnesium is involved in energy metabolism, enzyme activation, and the maintenance of normal muscle function. Phosphorus plays a key role in energy transfer processes and is an integral structural component of DNA, RNA, bones, and teeth [48]. Adequate intake of these minerals is particularly important for older adults, as aging is associated with an increased risk of hypertension, impaired muscle function, and reduced bone density—conditions that are closely linked to mineral status [49,50]. In the present study, the highest potassium content was determined in the cabbage core sample KS (2.85 ± 0.60 g/100 g dw). The steamed core sample (KT) exhibited a reduction of 0.33 g/100 g dw compared to the fresh core sample, suggesting minor mineral losses due to steaming. Similarly, Waseem et al. [24] reported potassium contents of 286 mg/100 g dw in the fresh cabbage and 279 mg/100 g dw in blanched cabbage, indicating only slight decreases after thermal processing. The magnesium contents in the samples LS, KS, and KT did not differ significantly; however, sample LT showed a significantly lower magnesium content compared to LS. Comparable findings were reported by Martinez et al. [51], who found similar magnesium content in cabbage leaves and cores, with cabbages generally exhibiting lower magnesium content compared to other *Brassica* species. The highest phosphorus content was determined in the cabbage core samples KS (0.50 ± 0.11 g/100 g dw) and KT (0.47 ± 0.10 g/100 g dw). Leaf samples contained approximately 1.5 times less phosphorus than core samples, highlighting the core as a richer source of this mineral fraction. Overall, although steaming resulted in slight reductions in mineral content, these losses were not substantial, indicating that by-products from cabbage processing retain significant mineral value, even after thermal treatment.

Overall, total sugar content was higher in leaf samples LS and LT (Table 6). In leaf samples, glucose was the predominant sugar, followed by fructose and sucrose. In contrast, sucrose was the dominant sugar in the core samples, with fructose and glucose being present in lower concentrations. No significant differences in fructose content were observed between the fresh (LS) and steamed (LT) leaf samples. However, in the core samples (KS and KT), fructose contents were significantly lower than those determined in the leaf samples. Rosa et al. [52] reported that the fructose contents of different white cabbage varieties ranged from 5.08 to 13.09 g/100 g dw. Similarly, Bhandari et al. [53] found fructose contents between 6.95 and 25.30 g/100 g dw. Both studies demonstrated considerable variability, largely attributed to differences in growing conditions and seasonal effects on sugar accumulation in cabbage. Compared with these published ranges, the fructose contents obtained in the present study were significantly higher. In general, compositional analyses of cabbage and related *Brassica* species show that glucose and fructose are typically the predominant free sugars, whereas sucrose represents a smaller portion of the total sugar content [54].

The glucose content of cabbage core samples (KS and KT) was, on average, three times lower than that of the respective cabbage leaf samples (LS and LT). Within the core samples, the fresh cores (KS) contained 1.5 times more glucose than steamed cores (KT), indicating that heat treatment (steaming) reduced glucose content in cabbage. Similar ranges for cabbage glucose content have been reported in the literature, with Bhandari et al. [53] reporting 11.58–27.67 g/100 g dw and Rosa et al. [52] reporting 3.10–9.62 g/100 g dw.

Maltose content was the highest in the steamed leaf sample (LT, 2.84 ± 0.23 g/100 g dw) and steamed core sample (KT, 2.43 ± 0.44 g/100 g dw), while the lowest value was observed in the fresh core sample (KS, 0.22 ± 0.05 g/100 g dw). This demonstrates that steaming significantly increased maltose content, with KT showing an 11-fold higher maltose content compared to KS.

Sucrose contents were greater in leaf samples LS (16.16 ± 3.28 g/100 g dw) and LT (15.33 ± 1.22 g/100 g dw), exceeding those of core samples KS and KT by more than twofold. Previous studies reported wide variability in cabbage’s sucrose content, ranging from 4.2−77.9 mg/g dw [53] and 5.6−17.8 mg/g dw [52]. The marked differences in sugar composition of leaves and cores likely reflect tissue-specific metabolic roles: cores primarily function as structural and transport tissues, explaining the predominance of sucrose as a translocated storage and transport sugar, whereas leaves, being photosynthetically active, accumulate higher contents of glucose, fructose, and total soluble sugars derived from carbon assimilation [55].

Steaming affected the total sugar content in leaf samples, while core samples (KS, 50.34 g/100 g dw, and KT, 49.60 g/100 g dw) showed only minor changes. Rosa et al. [52] found that the total sugar contents in cabbage ranged from 102.9 to 229.2 mg/g dw, while Bhandari et al. [53] reported 233.1–534.8 mg/g dw. The reduction in total sugars in steamed leaves suggested that thermal processing can induce sugar degradation, leaching, or structural transformation [56]. Steaming may promote sucrose hydrolysis into glucose and fructose and enhance thermal degradation of labile monosaccharides. In addition, heat can disrupt cell walls and membranes, facilitating the diffusion and partial loss of soluble sugars. The relatively minor changes observed in total sugar content observed in cores may be due to their denser tissue structure, which limits mass transfer and thermal damage.

Overall, the data indicate that leaf samples (LS and LT) contained more fructose, glucose, and total sugars, whereas core samples (KS and KT) were richer in sucrose. Maltose contents increased in all steamed cabbage samples, consistent with the known effects of steaming on *Brassica* carbohydrates. Thermal exposure disrupts plant tissue structure, enhances its accessibility, and promotes hydrolysis, resulting in significant changes in low-molecular-weight sugar profiles. These transformations are consistent with previous observations of heat-induced transformations of carbohydrate composition in cabbage and other *Brassica* vegetables [57].

#### 3.1.3. Total Phenolic Content and Antioxidant Activity of Cabbage Powders

The total phenolic contents (Table 7) differed significantly among all cabbage samples (*p* < 0.01). The highest total phenolic content was found in freeze-dried fresh cabbage cores (687.36 ± 2.12 mg GAE/100 g dw). Steamed cabbage samples exhibited lower phenolic content than fresh samples. This reduction can be explained by the fact that during steaming, cores and leaves are exposed to elevated temperatures, which may promote degradation of thermolabile phenolic compounds, including kaempferol or quercetin derivatives [58].

The total phenolic content and antiradical activity, measured using the DPPH and ABTS assays, indicate the antioxidant potential of the samples and their ability to neutralize free radicals, thereby reducing oxidative stress in the body. Antioxidants help protect cells from damage and prevent inflammation and the development of chronic diseases [59]. This is particularly important for older adults, as aging is associated with increased oxidative stress and a higher risk of degenerative and chronic conditions. Given the health-promoting effects of these compounds, this study evaluated their content in freeze-dried cabbage powders and examined how different processing methods influenced their contents.

All cabbage samples exhibited low-to-moderate DPPH antiradical activity. In contrast, Bas-Bellver et al. [37] reported DPPH values of 1.85 mg TE/g dw for freeze-dried cabbage and 2.08 mg TE/g dw for fresh cabbage, which are lower than the results obtained in the present study. This difference can be attributed to the relatively low content of lipid-soluble antioxidants in cabbage, which also contribute to DPPH activity. Significant differences were observed among all cabbage samples (*p* < 0.01). The highest DPPH antioxidant activity was found in the freeze-dried fresh core sample (14.03 ± 0.75 mg TE/100 g dw) and fresh leaf sample (13.59 ± 0.92 mg TE/100 g dw), followed by the freeze-dried powders made from steamed leaves and cores.

The ABTS antiradical activity of all cabbage samples was higher than previously reported, where fresh and freeze-dried cabbage showed activities of 30 mg TE/g dw and 25 mg TE/g dw, respectively [37]. This higher activity may be attributed to the higher content of water-soluble antioxidants, including vitamin C. Significant differences were observed between the freeze-dried fresh cabbage samples (LS and KS) (*p* < 0.01), whereas no significant differences were detected between steamed leaf and core samples. Steaming increased antioxidant content in leaf samples, likely due to tissue disruption enhancing extractability, while a decrease was observed in cores, possibly resulting from the prolonged steaming required for denser tissues, leading to antioxidant degradation.

#### 3.1.4. Volatile Compounds in Cabbage Powders

**Figure 2:**
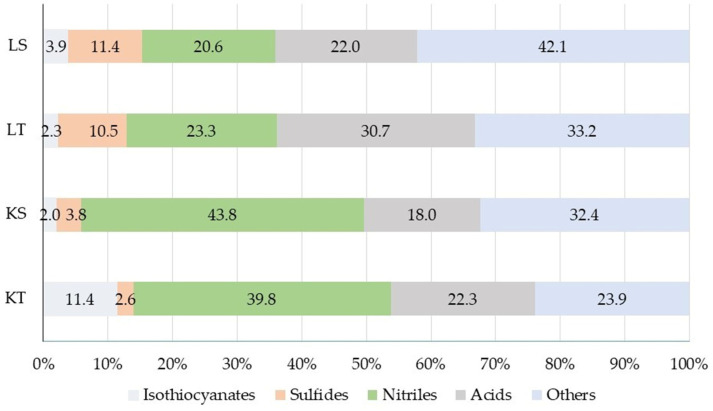
Volatile compound classes identified in cabbage processing by-product powders.

Volatile compounds grouped by chemical classes showed marked differences between the four cabbage powder samples (Figure 2).

Nitriles were the predominant class of volatile compounds identified in the core samples (KS and KT), accounting for 43.8% and 39.8%, respectively, indicating glucosinolate hydrolyses. In contrast, the proportion of nitriles in leaf samples was approximately twofold lower. Previous studies on *Brassica oleracea* varieties have shown that many of them preferentially release epithionitriles and nitriles rather than the biologically protective isothiocyanates during glucosinolate hydrolysis [60]. Our results are consistent with these reports, as substantial nitrile formation was also observed in the analyzed samples.

Isothiocyanates were most abundant in the KT sample, while their levels were significantly lower in other samples. Among glucosinolate-derived volatiles, isothiocyanates are particularly important, as they contribute to the characteristic pungent aroma and bitter taste of *Brassica* cultivars [60], and they are recognized for their health-promoting potential [61]. These compounds exhibit anti-inflammatory and antioxidant properties that may be especially beneficial for older adults by mitigating age-related oxidative stress and chronic inflammation.

A higher proportion of sulfides was detected in leaf samples. The dominant sulfide compound in leaves was dimethyl trisulfide, which is associated with cabbage-, garlic-, metallic-, and onion-like notes and is often described as an off-flavor [62]. The remaining volatile compounds (23.9−42.1%) consisted mainly of alcohols, ketones, and aldehydes. As reported by Wieczorek and Jelen [63], the most important volatile compounds in *Brassica* cultivars include sulfur compounds, nitriles, aldehydes and alcohols.

### 3.2. Description of Fiber-Enriched Jellies’ Quality AttributesDepending on Formulation

The developed cabbage powders are suitable for incorporation into various food products. In the present study, jelly intended for senior consumers was evaluated as a representative example. The development of the jelly formulation was carried out using a central composite design, which generated response surfaces based on two variables: cabbage powder and pectin concentrations (Table S1, Supplementary Materials). Descriptive statistics of responses are presented in Table 8.

**Figure 3:**
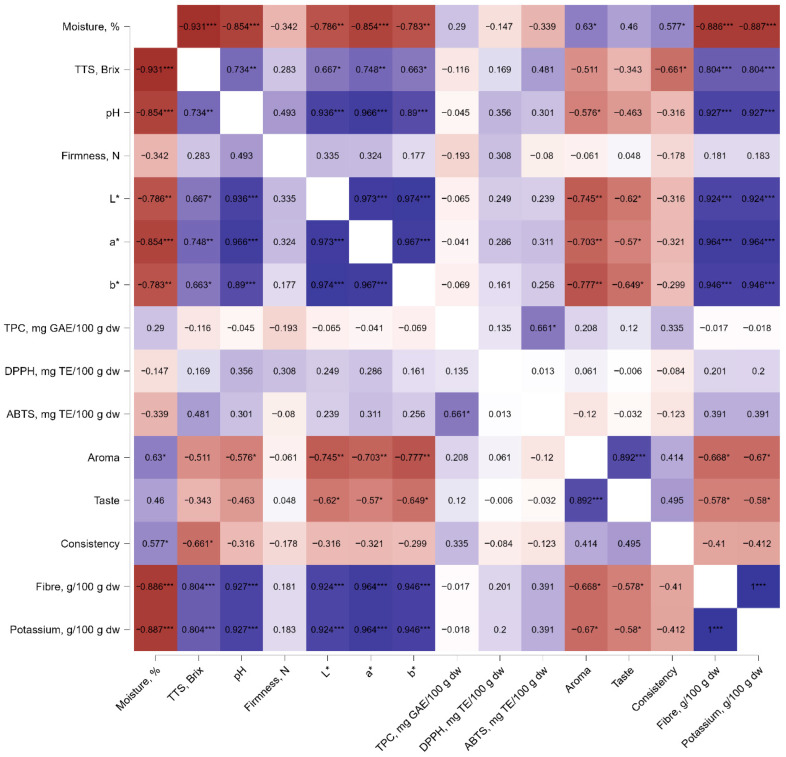
Pearson correlation heatmap showing relationships among physicochemical parameters (moisture, TSS, pH, firmness, L*, a*, b*), bioactive compounds (TPC), antioxidant activities (DPPH and ABTS), sensory attributes (aroma, taste, consistency), and nutritional components (fiber and potassium). Correlation coefficients (r) are displayed within each cell, with color intensity indicating the strength and direction of the correlations (blue = positive, red = negative). Asterisks indicate statistical significance (* *p* < 0.05, ** *p* < 0.01, *** *p* < 0.001).

The dataset formed five logical, interpretable clusters: physicochemical attributes (color, TTS, pH), moisture, firmness, antioxidant activity, and sensory properties (Figure S2, Supplementary Materials). TPC and ABTS antioxidant activity were moderately interrelated (Figure 3), while their correlations with other physical and sensory traits were weak. While many studies report strong correlations between total phenolic content (TPC) and ABTS/DPPH antioxidant assays, the strength of these relationships can vary depending on sample composition and phenolic profile. Moderate correlations have been documented, highlighting that other compounds or compositional factors can also influence antioxidant responses and that phenolic content is not the sole determinant of antioxidant activity [64].

The physicochemical attribute cluster, which includes the color components L*, a*, and b*, as well as pH, total soluble solids, was strongly associated with Factor A (cabbage powder concentration). This indicates that Factor A has a significant effect on these variables. A strong negative correlation with moisture was also observed—a decrease in moisture was accompanied by increases in the values of the color and chemical composition parameters.

**Figure 4:**
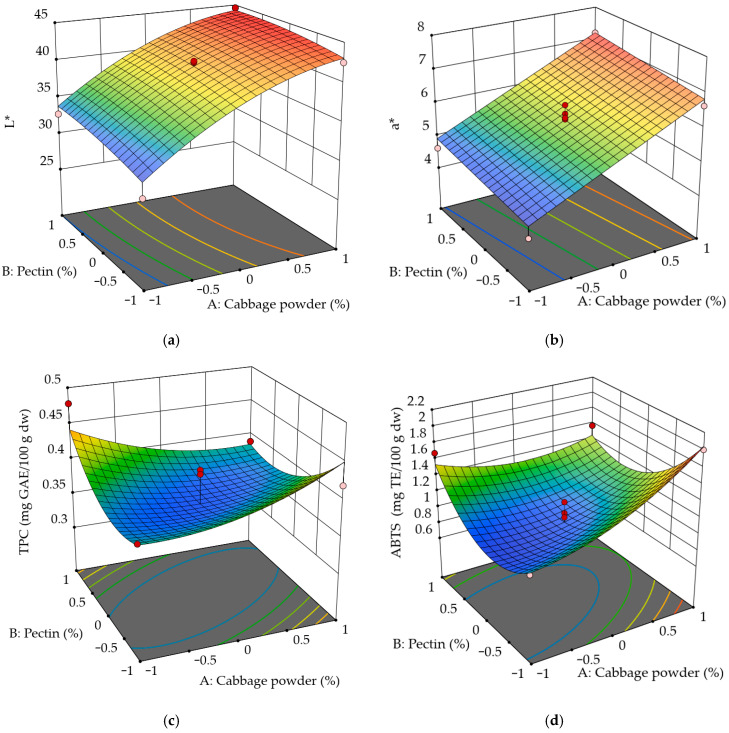

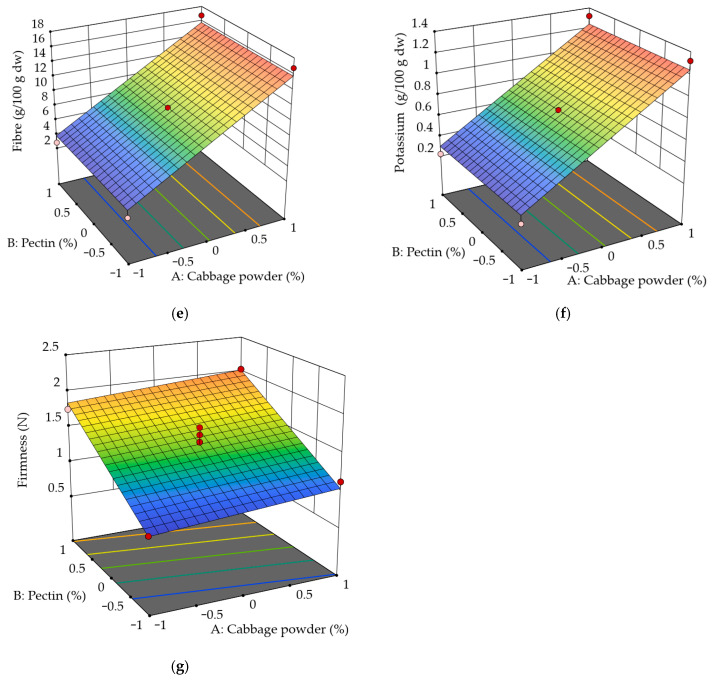
Response surface for color component L* (**a**), color component a* (**b**), total phenol content (**c**), ABTS antioxidant activity (**d**), fiber content (**e**), potassium content (**f**), and firmness (**g**), showing the effects of both cabbage powder and pectin concentrations.

Sensory evaluation of jellies incorporating cabbage processing by-products (cabbage powder) demonstrated that hedonic ratings for aroma, taste, and texture among the samples scored between 3 and 5 on a 5-point hedonic scale (3 = neither like nor dislike; 5 = like very much). A subset of panelists reported a perceptible cabbage aroma, but this attribute did not significantly influence overall liking. Panelists also noted a pleasant quince-like aroma; several panelists suggested reducing sweetness. Moisture, total soluble solids, pH, color component b*, DPPH antiradical activity, and all sensory parameters (aroma, taste, and texture) demonstrated low variation across the studied factor ranges (Table 8). Therefore, these variables were excluded from the modeling. The response surface for the L* color component (Figure 4a) demonstrates a non-linear relationship with cabbage powder concentration in the jelly formulation. Pectin concentration had no significant influence on L* value. As the concentration of cabbage powder increases, the L* value rises, indicating that the jelly color becomes lighter in color and the color intensity decreases. The present findings corroborate other studies on jellies that are enriched with fruit or vegetable powders, indicating that color parameters, particularly L*, are strongly influenced by powder concentration, with higher levels altering lightness and overall color perception [65]. Research on watermelon pomace-supplemented jellies confirmed that increasing powder addition modifies lightness and total color differences, demonstrating that the ingredient concentration can be used to target specific color outcomes [6]. In contrast to L*, color component a* displayed a linear relationship with cabbage concentration (Figure 4b).

The response surface for total phenolic content (TPC) (Figure 4c) revealed a non-linear, quadratic relationship with both cabbage powder concentration and pectin concentration. The surface exhibited a clear U-shaped pattern, indicating that the TPC did not change in a simple linear manner within the studied formulation range. At intermediate levels of both cabbage powder and pectin, the TPC was at a minimum, whereas lower or higher concentrations of these ingredients are associated with increased TPC. This behavior suggests the presence of significant second-order effects between formulation factors. Such non-linear responses of phenolic compounds are well documented in response surface methodology (RSM) studies, where second-order polynomial models incorporating linear, quadratic, and interaction terms are commonly required to adequately describe changes in total phenolic content. Previous optimization studies have demonstrated that phenolic responses frequently exhibit curvature, with local minima or maxima occurring at intermediate factor levels rather than following strictly linear trends [66]. The theoretical basis of RSM supports the use of quadratic models to capture both curvature and interactions between formulation variables, particularly for complex bioactive compounds such as phenolics [67]. In contrast to moisture and total soluble solids, which showed predominantly linear responses, TPC displayed a distinctly non-linear dependence on formulation changes, underscoring the complexity of phenolic behavior in jelly systems. Since ABTS antiradical activity was moderately correlated with TPC, the behavior pattern was similar, demonstrating a non-linear quadratic relationship with both cabbage powder and pectin concentrations (Figure 4d).

The response surface analysis showed a strong positive correlation between fiber content and cabbage powder concentration (Figure 4e), whereas the pectin concentration had no significant effect. A similar trend was observed for potassium content (Figure 4f), with increasing cabbage powder concentrations leading to higher potassium content, while pectin had a minimal influence. In contrast, firmness was primarily affected by the pectin concentration (Figure 4g). Overall, the responses for fiber content, potassium content, and firmness exhibited predominantly linear relationships with cabbage or pectin concentrations. Similar results were obtained by Yu et al. [68], who reported a significant effect of vegetable powders on the hardness of jellies. Studies on apple jelly formulation confirm that the pectin concentration chiefly influences textural properties such as gel firmness and elasticity, while juice or powder proportions predominantly affect soluble solids and color, supporting our observation that pectin affects firmness more than moisture or soluble solids [6,64]. Additionally, pectin’s interaction with water in food gels can influence texture, but often has a secondary effect on overall moisture content compared with the influence of solid ingredients [69].

The coded terms A (cabbage powder concentration) and B (pectin concentration) were fitted into equations:

L* = 40.62 + 5.47A + 0.83B − 0.23AB − 1.98A^2^ − 0.45B^2^

a* = 5.87 + 1.15A + 0.19B

ABTS = 0.98 + 0.20A − 0.05B − 0.24AB + 0.17A^2^ − 0.45B^2^

Fiber (g/100 g) = 9.77 + 5.99A

Potassium (g/100 g) = 0.77 + 0.47A

Firmness (N) = 1.43 + 0.09A + 0.49B

Model validation demonstrated R^2^ values above 0.9 for all variables except TPC and ABTS (Table 9).

The optimized response surface models showed that cabbage powder concentration (Factor A) was the dominant factor influencing the physicochemical characteristics of the jellies. Increases in Factor A led to marked increases in fiber and potassium content, indicating a strong compositional enrichment effect. Several responses, such as color attribute L* and ABTS, displayed significant quadratic behavior, suggesting nonlinear trends and the presence of optimal intermediate concentrations for achieving desirable visual quality. In contrast, Factor B exerted a more modest influence, contributing primarily to increased firmness and exerting a smaller effect on lightness. Interaction terms (AB) were generally minor but were relevant for color component a* and ABTS, where combined changes in both factors produced stronger effects than linear contributions alone. Together, these results demonstrate that product quality can be effectively managed by adjusting the level of cabbage powder to balance color properties and nutritional enhancement, while Factor B provides additional control over textural characteristics. Similar studies using food by-products in jellies have shown comparable potential, such as the incorporation of black carrot pomace and olive oil [70], watermelon pomace [6], and various fruit and vegetable powders [65].

Multi-response analysis identified an optimal formulation containing 3.08% cabbage powder and 2.5% pectin. The predicted responses for this formulation were 1.19 N firmness, L* and a* values of 40 and 5.86, respectively, TPC of 0.40 mg GAE/100 g dw, fiber content of 8.7 g/100 g dw, and potassium content of 0.68 g/100 g dw. Overall, this optimized formulation demonstrates a favorable balance between physicochemical quality, nutritional value, and sensory attributes, supporting its potential application in functional food development.

### 3.3. Study Limitations

This study has several limitations that should be acknowledged. First, the number of consumers participating in the hedonic test (n = 37) was relatively limited, particularly given that nine formulations were evaluated. Although this sample size may be acceptable for a preliminary assessment within a clearly defined target group (seniors aged 65+), it may not provide sufficient statistical power to detect small differences among samples or to support broad generalization of the findings. According to the International Organization for Standardization guideline ISO 11136 [71], larger consumer panels are generally recommended to ensure robust and reliable conclusions, particularly for market-oriented decision-making. Therefore, the present results should be interpreted as indicative of general trends rather than definitive evidence of consumer preference.

Second, because each consumer evaluated multiple samples, the data may have a repeated-measures structure, and a relatively small panel size may limit the stability and reliability of variance component estimates. For this reason, sensory data were not incorporated into predictive modeling and were instead used to provide supportive, descriptive insights.

An additional limitation concerns the scope of instrumental texture characterization. Although mechanical texture attributes such as hardness, springiness, and chewiness are commonly reported as key quality indicators for gel-based products, the present study included only a single-compression test, from which the maximum force (interpreted as firmness) was obtained. Given the soft, spoonable consistency of the developed jelly, parameters derived from multi-cycle compression tests (e.g., springiness and chewiness) were considered less representative of the intended mode of consumption, which does not involve mastication or elastic recovery during repeated bites. Nevertheless, restricting the analysis to a single instrumental parameter may not fully capture other relevant aspects of structural behavior, such as viscoelastic properties or structural resilience, that could influence consumer perception.

## 4. Conclusions

Steaming of cabbage leaves and cores increased their water absorption capacity but reduced water solubility and negatively affected color, resulting in darker powders with more yellow–red hues due to pigment degradation and thermal reactions. Both leaf and core powders were nutritionally valuable, exhibiting high dietary fiber content (>30 g/100 g dw) and maintaining relevant vitamin C content. Mineral contents, especially potassium and phosphorus, were higher in cores and only moderately affected by steaming.

The developed cabbage powders were tested in a texture-modified jelly as an application case study. The optimized response surface models showed that the cabbage powder concentration was the dominant factor influencing the physicochemical and sensory characteristics of the jellies. A strong negative correlation with jelly’s moisture was also observed, where decreasing moisture corresponded to increases in the color intensity and chemical composition of products. These results suggest that jellies enriched with powders made from cabbage processing by-products can be effectively managed by adjusting the cabbage powder concentration to achieve an optimal balance between moisture reduction, color, and nutritional enhancement.

## Figures and Tables

**Table 1:** Two-factor central composite design for optimization of jelly formulation.

Standard Order	Run Order	Cabbage Powder Concentration, %	Pectin Concentration, %
13	1	3.5	2
7	2	3.5	1.293
1	3	1	1.5
8	4	3.5	2.707
3	5	0.965	2
12	6	3.5	2
5	7	3.5	2
2	8	6	1.5
6	9	6.035	2
10	10	3.5	2
11	11	3.5	2
4	12	6	2.5
9	13	3.5	2

**Table 2:** Chemical characteristics of Japanese quince syrup.

Variables	Quince Syrup *
pH	2.51 ± 0.01
Titratable acid, mg/g	0.39 ± 0.01
Soluble solids, °Brix	58.7 ± 0.1
Total phenolic content, mg GAE/100 g	0.38 ± 0.02
Antiradical activity (DPPH), mg TE/100 g	1.16 ± 0.13
Total carotenoids, mg/100 g	8.74 ± 1.1

* Data presented in the table are based on the manufacturer’s specifications.

**Table 3:** Water-related properties of cabbage powder samples.

Samples	Moisture Content, %	Water Activity a_w_	Water Absorption, %	Water Solubility Index, %
LS	13.79 ± 0.73 ^a^	0.195 ± 0.005 ^a^	4.22 ± 0.14 ^d^	55.24 ± 2.40 ^a^
LT	11.51 ± 0.22 ^b^	0.189 ± 0.001 ^ab^	5.44 ± 0.10 ^a^	46.97 ± 1.34 ^b^
KS	11.68 ± 0.31 ^b^	0.185 ± 0.003 ^b^	4.53 ± 0.02 ^c^	52.60 ± 0.53 ^a^
KT	9.47 ± 0.46 ^c^	0.143 ± 0.003 ^c^	4.83 ± 0.03 ^b^	44.06 ± 2.27 ^b^

^a–d^—different letters in the columns show that there is a significant difference between the values (*p* ˂ 0.01).

**Table 4:** Color of freeze-dried cabbage leaf and core powders.

Samples	L*	a*	b*	Appearance
LS	91.86 ± 0.27 ^a^	−1.28 ± 0.10 ^d^	13.47 ± 0.18 ^b^	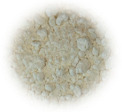
LT	87.79 ± 0.17 ^c^	−0.03 ± 0.07 ^b^	15.27 ± 0.37 ^a^	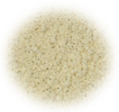
KS	91.38 ± 0.23 ^b^	−0.62 ± 0.19 ^c^	11.93 ± 0.24 ^c^	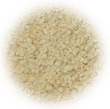
KT	87.80 ± 0.11 ^c^	0.21 ± 0.02 ^a^	14.71 ± 0.65 ^a^	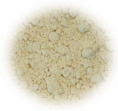

^a–d^—different letters in the columns show that there is a significant difference between the values (*p* ˂ 0.01).

**Table 5:** Nutritional value of freeze-dried cabbage leaf and core powders.

Samples	Dietary Fiber, g/100 g dw	Protein, g/100 g dw	Vitamin C, g/100 g dw	Minerals, g/100 g dw		
				Potassium	Magnesium	Phosphorus
LS	31.88 ± 0.22 ^c^	11.02 ± 1.16 ^b^	0.317 ± 0.001 ^a^	2.62 ± 0.55 ^a^	0.24 ± 0.05 ^a^	0.34 ± 0.07 ^b^
LT	32.89 ± 0.32 ^bc^	9.83 ± 1.02 ^c^	0.197 ± 0.011 ^b^	2.47 ± 0.52 ^a^	0.14 ± 0.02 ^b^	0.31 ± 0.07 ^b^
KS	33.70 ± 0.40 ^ba^	16.08 ± 1.25 ^a^	0.317 ± 0.011 ^a^	2.85 ± 0.60 ^a^	0.20 ± 0.03 ^a^	0.50 ± 0.11 ^a^
KT	34.75 ± 0.20 ^a^	15.91 ± 1.33 ^a^	0.184 ± 0.010 ^b^	2.52 ± 0.53 ^a^	0.21 ± 0.03 ^a^	0.47 ± 0.10 ^ab^

^a–c^—different letters in the columns show that there is a significant difference between the values (*p* ˂ 0.01).

**Table 6:** Sugar contents in cabbage powder samples, g/100 g dw.

Sample	Fructose	Galactose	Glucose	Maltose	Sucrose	Sum of Individual Sugars
LS	18.19 ± 3.62 ^a^	0.41 ± 0.08 ^a^	22.49 ± 4.52 ^a^	1.92 ± 0.34 ^b^	16.16 ± 3.28 ^b^	59.17 ± 6.66 ^a^
LT	17.93 ± 1.43 ^a^	0.43 ± 0.03 ^a^	21.74 ± 1.74 ^a^	2.84 ± 0.23 ^a^	15.33 ± 1.22 ^b^	58.27 ± 2.57 ^a^
KS	6.11 ± 1.25 ^b^	0.42 ± 0.08 ^a^	8.83 ± 1.81 ^b^	0.22 ± 0.05 ^c^	34.76 ± 6.91 ^a^	50.34 ± 7.25 ^b^
KT	5.30 ± 1.10 ^b^	n.d.	6.08 ± 1.22 ^b^	2.43 ± 0.44 ^a^	35.79 ± 7.18 ^a^	49.60 ± 7.38 ^b^

^a–^^c^—different letters in the columns show that there is a significant difference between the values (*p* ˂ 0.05). n.d.—not detected (outside the test range).

**Table 7:** Total phenolic contents and antiradical activity of cabbage samples.

Sample	Total Phenolic Content, mg GAE/100 g dw	Antiradical Activity (DPPH), mg TE/100 g dw	Antiradical Activity (ABTS), mg TE/100 g dw
LS	614.3 ± 31.4 ^b^	13.59 ± 0.92 ^a^	25.95 ± 1.40 ^c^
LT	554.2 ± 21.2 ^c^	12.13 ± 0.32 ^b^	33.01 ± 1.79 ^b^
KS	687.4 ± 12.1 ^a^	14.03 ± 0.75 ^a^	42.48 ± 2.55 ^a^
KT	491.8 ± 24.1 ^d^	10.38 ± 0.42 ^c^	35.18 ± 1.98 ^b^

^a–d^—different letters in the columns show that there is a significant difference between the values (*p* ˂ 0.01).

**Table 8:** Descriptive statistics of response variables obtained from the central composite design for jelly formulation development.

Responses	Min	Max	Median	Mean	Std. Deviation	Variance	CV%
Moisture, %	53.69	57.28	55.43	55.68	1.16	1.337	2.08
TSS, Brix	39.40	43.20	41.15	41.21	1.14	1.309	2.78
pH	3.297	3.707	3.54	3.53	0.12	0.014	3.34
Firmness, N	0.70	2.21	1.46	1.43	0.43	0.183	29.85
L*	29.70	44.59	40.45	39.12	4.87	23.7	12.45
a*	4.18	7.30	5.92	5.87	0.97	0.949	16.60
b*	19.71	27.05	24.65	24.00	2.40	5.742	9.98
TPC, mg GAE/100 g dw	0.334	0.478	0.399	0.396	0.044	0.002	11.11
DPPH, mg TE/100 g dw	0.755	0.951	0.847	0.843	0.069	0.005	8.19
ABTS, mg TE/100 g dw	0.706	2.003	1.230	1.327	0.410	0.168	30.90
Aroma	3.30	3.81	3.51	3.52	0.15	0.021	4.15
Taste	3.46	4.05	3.81	3.78	0.17	0.029	4.47
Texture	3.68	4.03	3.95	3.90	0.10	0.011	2.64
Fiber, g/100 g dw	2.69	16.85	9.77	9.77	4.96	24.59	50.76
Potassium, g/100 g dw	0.21	1.32	0.77	0.77	0.39	0.152	50.72

**Table 9:** Fit statistics for response surface models.

Responses	F-Value	*p*-Value	R^2^	Significant Model Terms
Firmness	51.05	<0.0001	0.9108	B
L	31.45	0.0155	0.9572	A, A^2^
a	97.46	<0.0001	0.9512	AB
TPC	1.57	0.0398	0.5282	-
ABTS	9.89	0.002	0.870	A, AB, B^2^
Fiber	184.41	<0.0001	0.9417	A
Potassium	178.75	<0.0001	0.9728	A

## Data Availability

The original contributions presented in this study are included in the article/Supplementary Materials. Further inquiries can be directed to the corresponding author.

## Supplementary Materials

The following supporting information can be downloaded at https://www.mdpi.com/article/10.3390/foods15061009/s1: Figure S1: Particle size distribution in cabbage by-product powders: freeze dried fresh leaves (a); freeze-dried steamed leaves (b); freeze-dried fresh cores (c); freeze-dried steamed cores (d); Figure S2. Correlation network diagram: nodes–variables; green edges–strong positive correlations; red edges–strong negative correlations; Table S1. Responses obtained in central composite design for jelly formulation development.

## Abbreviations

The following abbreviations are used in this manuscript:

LS	Freeze-dried fresh cabbage leaves
LT	Freeze-dried steamed cabbage leaves
KS	Freeze-dried fresh cabbage cores
KT	Freeze-dried steamed cabbage cores
ABTS	2,2′-azino-bis(3-ethylbenz-thiazoline-6-sulfonic) acid
ANOVA	Analysis of variance
DPPH	2,2-diphenyl-1-picrylhydrazyl
GAE	Gallic acid equivalents
n.d.	Not detected (outside the test range)
RSM	Response surface methodology
TE	Trolox equivalent
TPC	Total phenolic content
TTS	Total soluble solids
WAI	Water absorption index
WSI	Water solubility index
