# Supplementary material for: Valorization of By-Products from White Cabbage (Brassica oleracea var. capitata) Processing
Source: Foods. 2026 Mar 12;15(6):1009. doi: 10.3390/foods15061009 (PMC13024762; doi:10.3390/foods15061009)
Supplement: Supplementary file 1 [file foods-15-01009-s001.zip › Figure S1 particle size distribution.pdf]

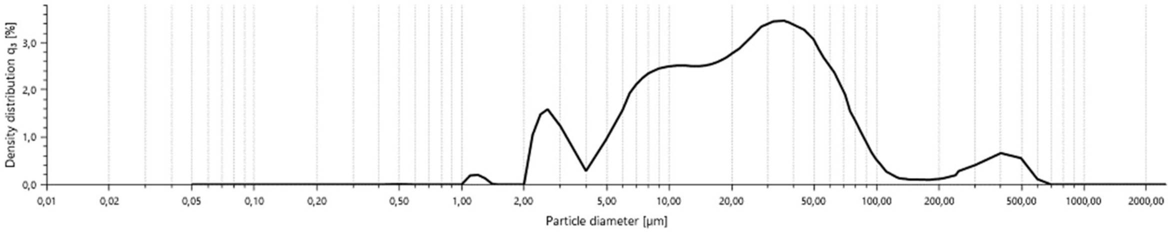

(a)

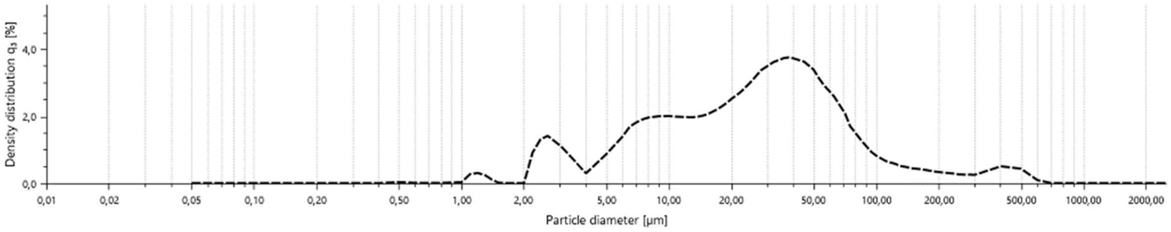

(b)

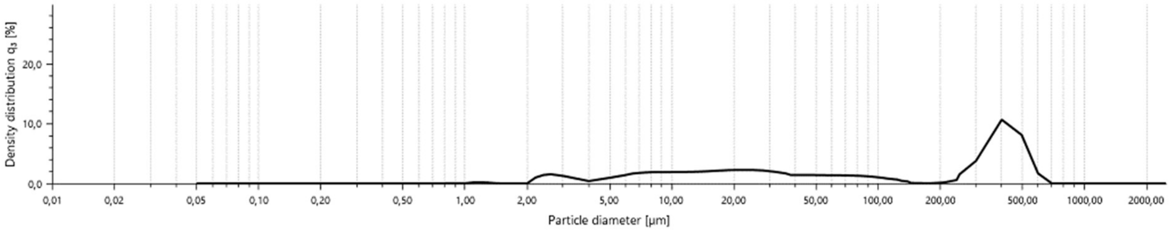

(c)

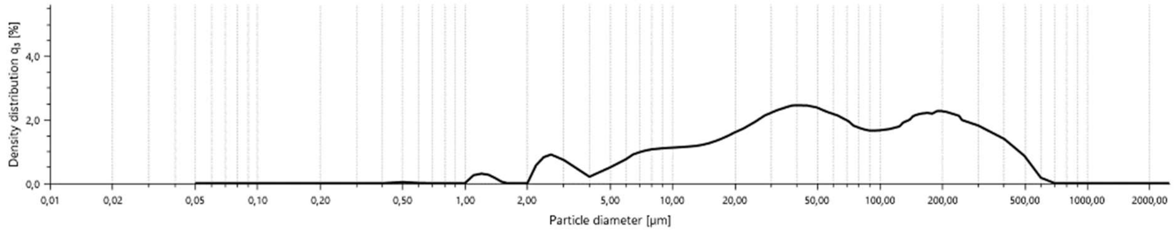

(d)

Figure S1. Particle size distribution in cabbage by-product powders: freeze dried fresh leaves (a); freeze-dried steamed leaves (b); freeze-dried fresh cores (c); freeze-dried steamed cores (d).
