# Supplementary material for: Valorization of By-Products from White Cabbage (Brassica oleracea var. capitata) Processing
Source: Foods. 2026 Mar 12;15(6):1009. doi: 10.3390/foods15061009 (PMC13024762; doi:10.3390/foods15061009)
Supplement: Supplementary file 1 [file foods-15-01009-s001.zip › Figure S2 Correlation network.pdf]

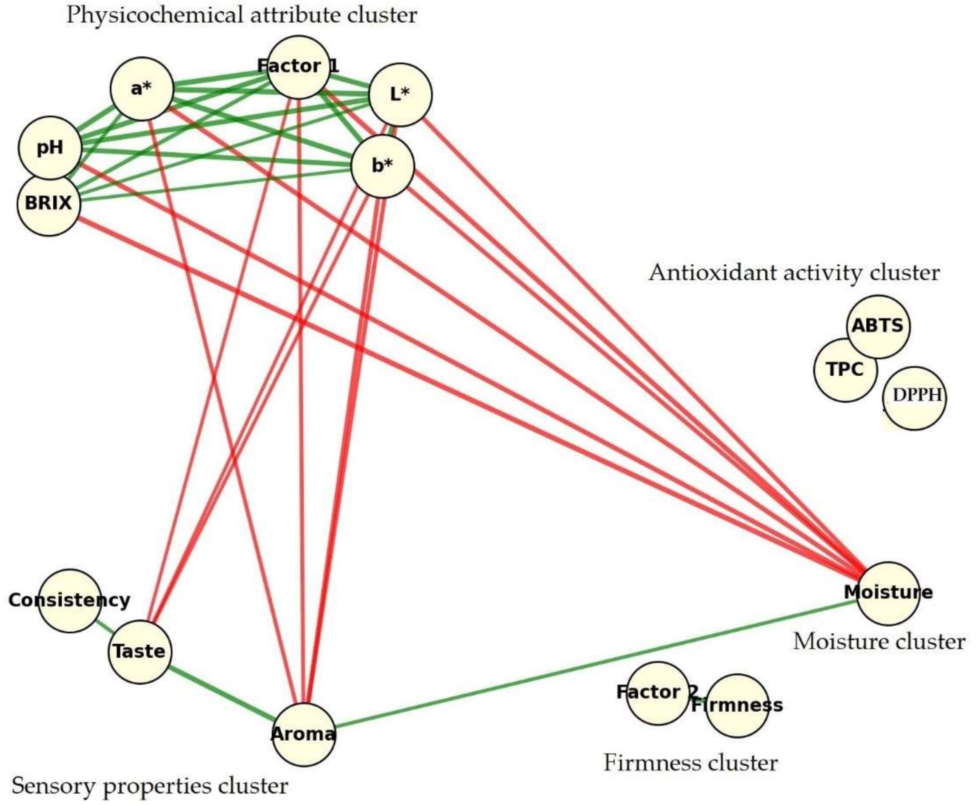

Figure S2. Correlation network diagram: nodes – variables; green edges – strong positive correlations; red edges – strong negative correlations.
