# Supplementary material for: Valorization of By-Products from White Cabbage (Brassica oleracea var. capitata) Processing
Source: Foods. 2026 Mar 12;15(6):1009. doi: 10.3390/foods15061009 (PMC13024762; doi:10.3390/foods15061009)
Supplement: Supplementary file 1 [file foods-15-01009-s001.zip › Table S1 Responses.pdf]

Table S1. Responses obtained in central composite design for jelly formulation development

Run number	Factor A	Factor B	Moisture, %	TTS, Brix	pH	Firmness, N	L*	a*	b*	TPC, mg GAE/100 g dw	DPPH, mg TE/100 g dw	ABTS, mg TE/100 g dw	Aroma	Taste	Consistency	Fibre, g/100 g dw	Potassium, g/100 g dw
1	0	0	55.35	41.85	3.523	1.66	40.45	5.92	24.65	0.337	0.908	1.039	3.49	3.76	3.74	9.77	0.77
2	0	-1.4142	56.44	40.55	3.466	0.70	39.98	5.78	24.83	0.455	0.768	1.905	3.38	3.57	3.92	9.77	0.77
3	-1	-1	56.76	40.55	3.297	0.86	29.70	4.18	20.22	0.388	0.755	1.127	3.59	3.86	3.95	2.79	0.22
4	0	1.4142	55.43	41.15	3.598	2.21	41.06	6.16	24.62	0.399	0.765	1.532	3.54	3.95	4.03	9.77	0.77
5	-1	1	57.18	40.50	3.432	1.75	32.71	4.61	19.71	0.478	0.951	1.676	3.81	4.05	3.95	2.79	0.22
6	0	0	54.98	41.00	3.574	1.56	40.05	6.04	24.08	0.334	0.905	1.092	3.41	3.68	3.92	9.77	0.77
7	-1.4142	0	57.28	39.40	3.367	1.16	30.73	4.37	20.37	0.374	0.787	0.975	3.78	4	3.95	2.69	0.21
8	1	-1	54.10	43.00	3.633	1.12	42.51	6.63	25.99	0.419	0.821	2.003	3.51	3.81	3.81	16.75	1.32
9	1.4142	0	54.73	42.05	3.688	1.28	44.19	7.30	27.05	0.425	0.941	1.558	3.54	3.81	4	16.85	1.32
10	0	0	55.31	41.55	3.536	1.47	40.16	5.88	23.93	0.336	0.783	0.830	3.55	3.82	3.87	9.77	0.77
11	0	0	55.77	41.35	3.540	1.38	41.30	6.30	25.18	0.406	0.860	1.230	3.39	3.76	3.95	9.77	0.77
12	1	1	53.69	43.20	3.707	2.03	44.59	7.20	26.49	0.389	0.847	1.573	3.30	3.46	3.68	16.75	1.32
13	0	0	56.83	39.60	3.544	1.46	41.13	5.89	24.82	0.412	0.867	0.706	3.49	3.62	3.98	9.77	0.77

Shaded columns indicate variables selected for model optimization.
